# Integrative Analysis of m6A RNA Methylation Regulators and the Tumor Immune Microenvironment in Non-Small-Cell Lung Cancer

**DOI:** 10.1155/2022/2989200

**Published:** 2022-02-09

**Authors:** Jiaqi Zhu, Yun Jiang, Tianyi Wang, Anqi Wu, Tingting Zhou, Anping Zhang, Yijie Tang, Zihao Shen, Jinjie Wang, Hao Zhou, Jiahai Shi, Jianle Chen

**Affiliations:** ^1^Nantong Key Laboratory of Translational Medicine in Cardiothoracic Diseases and Research Institution of Translational Medicine in Cardiothoracic Diseases, Affiliated Hospital of Nantong University, No. 20, Xisi Road, Nantong, 226001 Jiangsu, China; ^2^Department of Thoracic Surgery, Affiliated Hospital of Nantong University, No. 20, Xisi Road, Nantong, 226001 Jiangsu, China

## Abstract

**Background:**

Non-small-cell lung cancer (NSCLC) is a major component of lung cancer and is significantly correlated with poor prognosis. N6-methyladenosine (m6A) RNA methylation is closely related to the occurrence, progression, and prognosis of cancer. The potential biological functions and mechanisms of m6A RNA methylation in the immune microenvironment are still unclear.

**Methods:**

We assessed m6A RNA methylation modification patterns in 1326 NSCLC patient samples based on 20 m6A regulators, linking these clusters to the tumor microenvironment and immune cell infiltration. The m6Ascore was created to quantify the m6A modification patterns of individual tumors. We then assessed the value of NSCLC patients in terms of clinical prognosis and immunotherapy response.

**Results:**

According to different mRNA expression levels, two different m6A clusters were identified. m6A aggregation was significantly associated with clinical prognostic characteristics, the tumor microenvironment, and immune-related biological processes. Fifteen differential genes were screened based on these two m6A clusters, and to further investigate the mechanisms of action of these differential genes, they were subjected to unsupervised clustering analysis, which classified them into four different genomic isoforms. Prognostic analysis indicated that the survival advantage of the m6A gene cluster A modification mode was significantly prominent. We continued to construct the m6Ascore, which was used as a scoring tool to evaluate tumor typing, immunity, and prognosis. Patients with a low m6Ascore showed a significant survival advantage, and the group with a low m6Ascore had a better prognosis predicted by immunotherapy. The anti-PD-1/L1 immunotherapy cohort showed that a lower m6Ascore was associated with higher efficacy of immunotherapy.

**Conclusions:**

The results suggest that m6A RNA methylation regulators make an important difference in the tumor immune microenvironment of patients with NSCLC. m6A gene characterization and the construction of the m6Ascore provide us with a richer understanding of m6A RNA methylation modification patterns in NSCLC patients and help to predict clinical prognosis and immunotherapeutic response.

## 1. Introduction

Lung cancer is currently one of the most common malignant tumors in the world. It has a very high mortality rate, and the 5-year survival rate is only 16%. According to the latest cancer statistics, it is estimated that in 2020 alone, there will be nearly 20 million new cancer cases and 10 million tumor deaths worldwide, with lung cancer patients topping the list with a mortality rate of 18% [1, 2]. Lung cancer includes NSCLC and small-cell lung cancer, among which NSCLC accounts for the largest proportion of all lung cancer patients, approximately 85-90% [3]. In recent decades, with the continuous improvement of medical technology, a series of therapies, such as surgery, chemotherapy, radiotherapy, immunotherapy, and adjuvant Chinese medicine, have been used to treat patients with NSCLC; however, because of imperfect screening programs and late clinical symptoms, most patients are diagnosed with lung cancer at an advanced stage with poor prognosis [4, 5]. Thus, NSCLC has become a thorn in the side of oncologic diseases, and effective treatment regimens are needed to improve the currently dismal prospects for NSCLC outcomes.

Based on previous studies, m6A is the most frequently distributed form of mRNA modification in eukaryotes [6, 7] and has become the focus of scientific research in the past ten years. m6A methylation makes a large difference in cancer through multiple mechanisms [8] and is particularly important in the prediction of tumor development, immunotherapy, and outcome [9, 10]. m6A modification is a protein-mediated, dynamic, and reversible process. m6A regulators consist of three main protein types, namely, m6A methyltransferase (also known as writer), m6A demethylase (also known as eraser), and m6A binding protein (also known as reader) [11–14]. A variety of domestic and foreign studies indicate that m6A regulatory factors play an important role in a variety of biological functions and mechanisms in vivo [15], can regulate the TME, and can play a regulatory role in counteracting PD-L1 resistance [16, 17]. Dysregulation of m6A modification is significantly associated with malignant progression and abnormal immune regulation [18]. For example, METTL3-mediated m6A modification physiologically promotes activation of dendritic cell (DC) and DC-based T cell responses [19], and loss of YTHDF1 enhances CD8+ T cell tumor infiltration, making anti-PD-L1 therapy more effective [20]. To date, the specific mechanisms of m6A modification involved in NSCLC development and progression and the immune response are not well understood.

In this paper, we first collected data through The Cancer Genome Atlas (TCGA) database and Gene Expression Omnibus (GEO) to integrate information on mRNA and protein levels in NSCLC and assessed the impact of m6A regulator imbalance on NSCLC development and progression, tumor microenvironment, immune response, and prognosis. Then, we obtained two different methylation modification patterns, which were found to have different clinical characteristics, immune microenvironments, and prognostic values. We also developed a scoring system, called m6Ascore, to quantify the m6A modification patterns of individual patients and to determine their value in predicting prognosis and treatment response in NSCLC patients.

## 2. Materials and Methods

### 2.1. Data Acquisition

In this article, gene expression profiling data of NSCLC patients and clinically relevant data were obtained using TCGA [21] database, including FPKM values of gene expression from 1037 NSCLC samples and 108 normal samples, followed by the conversion of FPKM values into TPM values for data processing. Among further examinations, we deleted samples with no survival information. In addition, a study-eligible dataset (GSE50081) was collected in the GEO database, which included gene sequencing information and clinical information for 181 NSCLC case samples, and a standardized matrix file was downloaded for validation of TCGA data in the prognostic gene signature of TCGA data. In addition, mutation data were downloaded from TCGA database; m6A CNV data were obtained from UCSC Xena. All data processing was analyzed using R (version 4.0.4) and the R Bioconductor packages.

### 2.2. m6A Ribonucleic Acid Methylation Regulator Collection

Referring to the existing studies in the past, we extracted 20 m6A regulators for further study of different m6A modification patterns. These 20 m6A regulators included 6 writers (METTL3, METTL14, WTAP, ZC3H13, RBM15, and RBM15B), 12 readers (YTHDC1, YTHDC2, YTHDF1, YTHDF2, YTHDF3, HNRNPC, LRPPRC HNRNPA2B1, IGF2BP2, IGF2BP3, EIF3A, and EIF4E), and 2 erasers (FTO and ALKBH5) (Table 1) [11, 22]. A consistent clustering algorithm was then used for further analysis of patients [23]. To further explore the potential biological functions of m6A regulation, we performed this procedure using the R package “ConsensusClusterPlus,” which guarantees stability with 1000 replicates and an 80% resampling rate [24].

### 2.3. Gene Set Variance Analysis (GSVA)

We used the “GSVA,” “GSEABase,” and “Limma” R software packages for GSVA enrichment to further study biological differences in m6A modification patterns. We used GSVA, a GSE method that estimates pathway changes in the total sample size in an unsupervised manner [25]. C2. Cp. Kegg. V7.4. Symbols. GMT is a set of specific gene files, and we downloaded it from the MSigDB database for further analysis. An adjusted *P* value < 0.05 and FDR < 0.05 were considered to be statistically significant, and pathway heatmaps were plotted under this condition.

### 2.4. Inference of Tumor Microenvironment and Immune Cells

We used the ssGSEA algorithm to calculate the relative abundance of each immunoinfiltrating cell in NSCLC. We used the “Ggpubr” package for data analysis and the ggplot2 package for boxplotting. There were 23 types of immune cells evaluated by the ssGSEA algorithm, including activated B cells, activated CD4 T cells, activated dendritic cells, mast cells, eosinophils, natural killer cells, natural killer T cells, and neutrophils.

### 2.5. Identification of Differentially Expressed Genes (DEGs)

The “Limma” R package was applied to calculate the differences between different clusters. This method proved to be particularly beneficial in experiments with small sample sizes, ensuring reliable and stable inference even with a small number of replications [26]. When the adjusted *P* value of DEGs < 0.001, log_2_ fold change > 1, it was considered to be significant. We divided 20 m6A regulators into two different clusters based on their mRNA expression levels. GO and KEGG enrichment analyses were run using the “Clusterprofiler” R package to understand the pathways of action associated with DEGs. We screened for fifteen differential genes based on these two m6A clusters and further investigated the mechanisms of action of these differential genes. They were subjected to unsupervised clustering analysis, which classified them into four different genomic isoforms.

### 2.6. Construction of m6Ascores

We established a scoring system to assess m6A modification patterns in individual NSCLC patients. We called it the m6Ascore. The m6A scoring system was constructed in the following steps. First, DEGs screened from two m6A clusters were homogenized in all sample data to extract crossover genes. Then, univariate Cox regression analysis was performed for each gene, and the prognostic genes were extracted for further analysis. The m6A-related gene markers were constructed by PCA. PC1 and PC2 were selected as signature scores. The m6Ascore was calculated using the following equation:

(1) $$\begin{array}{c} \text{m}6\text{Ascore}=\sum \left(\text{PC}1i+\text{PC}2i\right), \end{array}$$

where *i* is the expression value of each selected gene [27].

### 2.7. Statistical Analysis

All data processing was performed in the R (4.0.5) statistical package. CNVs of 20 m6A regulators on different chromosomes were plotted using the “RCircos” R package. Student's *t*-test was used to assess the differences between two groups. The Kruskal–Wallis test was used to determine more groups for comparison of differences. Kaplan–Meier survival curves were used to analyze their prognostic value. The Wilcoxon test was performed when comparing differences between groups. We calculated risk ratios (HRs) for m6A regulators and associated genes in different clusters of m6A by univariate Cox regression models. The association between m6Ascores and age, sex, and pathological stage was assessed using chi-square tests. The “Maftools” R package was used to map the total mutations of m6A regulators and subtypes in TCGA-NSCLC cohort. All statistical values were bilateral, and *P* < 0.05 was considered to be statistically significant.

## 3. Results

### 3.1. Landscape of Genetic Variation in m6A Regulators in NSCLC

**Figure 1:**
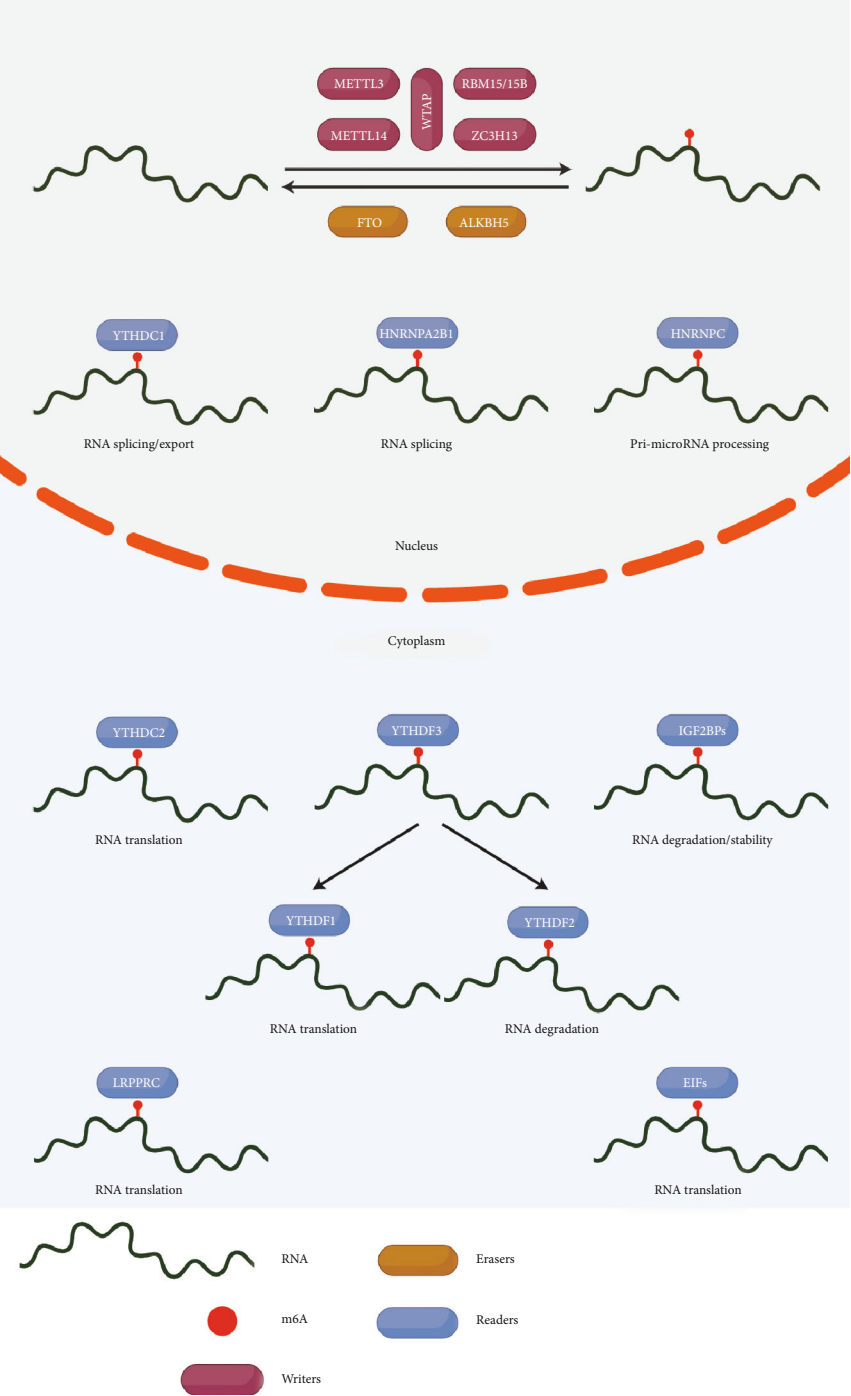
A flow chart summarizing the mechanisms of RNA methylation modification mediated by 20 m6A regulators and their biological functions on RNA.

**Figure 2:**
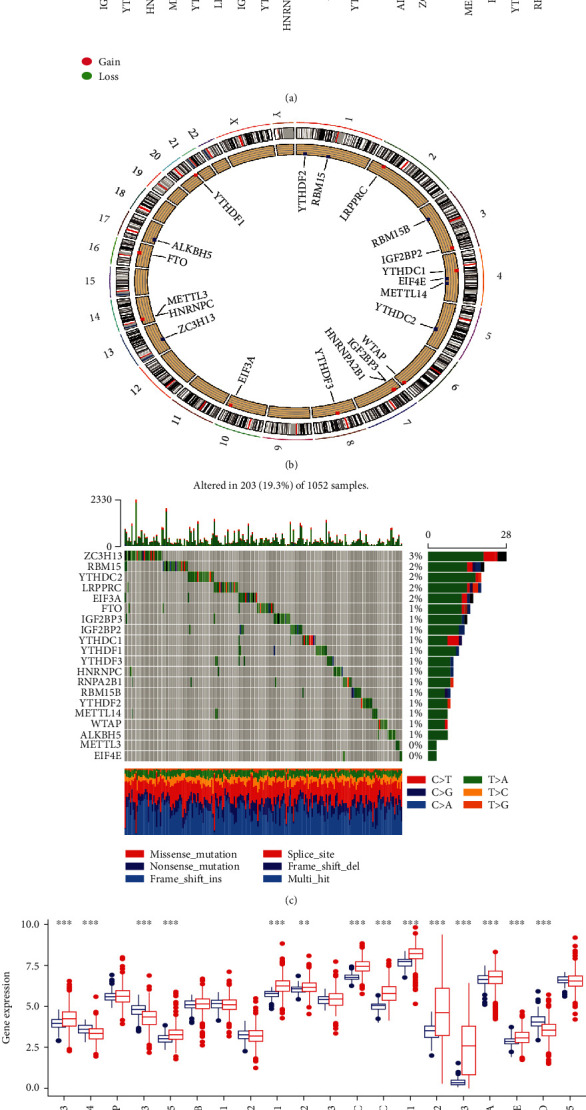
Landscape of genetic variation in m6A regulators in NSCLC. (a) The graph depicts the frequency of CNV alterations in 20 m6A regulators in NSCLC, with loss (gain) frequencies marked with green (red) dots. (b) Mutation sites of m6A regulators on 23 chromosomes. (c) Mutation frequency of m6A regulators in NSCLC, with each column representing an individual patient, the number and bar graph on the right representing mutation frequency of each regulator, and the stacked bar graph below representing transformation of each sample. (d) Regulation between m6A mRNA expression levels. Tumor, red; normal tissue, blue. The asterisk represents the *P* values (^∗^*P* < 0.05; ^∗∗^*P* < 0.01; ^∗∗∗^*P* < 0.001).

In the current study, we identified 20 m6A regulators based on previous research results, including 6 writers, 12 readers, and 2 erasers. The dynamic reversible processes of m6A RNA methylation modification mechanisms mediated by 20 m6A regulators and their potential biological functions on RNA are summarized in Figure 1. First, we integrated the somatic mutation and CNV data of 20 m6A regulators in NSCLC and calculated their incidence for further observation. We studied the CNV of the m6A regulator and confirmed its universality, mainly focusing on the high gain of varying frequency, such as YTHDC1, IGF2BP2, METTL3, YTHDF1, and HNRNPC. YTHDF2, ZC3H13, EIF4E, YTHDC2, ALKBH5 METTL14, and RBM15 showed a loss of alteration frequency in CNV alterations (Figure 2(a)). Mutation sites of the m6A regulatory factor on chromosomes are shown in Figure 2(b). Then, we found that the total mutation frequency of m6A regulators was low, and 203 out of 1052 populations were mutated, with a frequency of 19.3%. The highest mutation frequency was found in ZC3H13, with 3%, while METTL3 and EIF4E showed no mutations (Figure 2(c)). The expression levels of m6A regulators in NSCLC tumor samples and normal tissue samples were also studied, and it was found that 14 of the 20 m6A regulators were significantly and differentially expressed (Figure 2(d)). The above analysis indicated that m6A regulator variants and genetic expression were highly variable between NSCLC and normal tissues. These results indicate that the expression imbalance of m6A regulators plays a significant role in the occurrence and progression of NSCLC.

### 3.2. Patterns of m6A Modifications Mediated by 20 m6A Regulators in NSCLC

**Figure 3:**
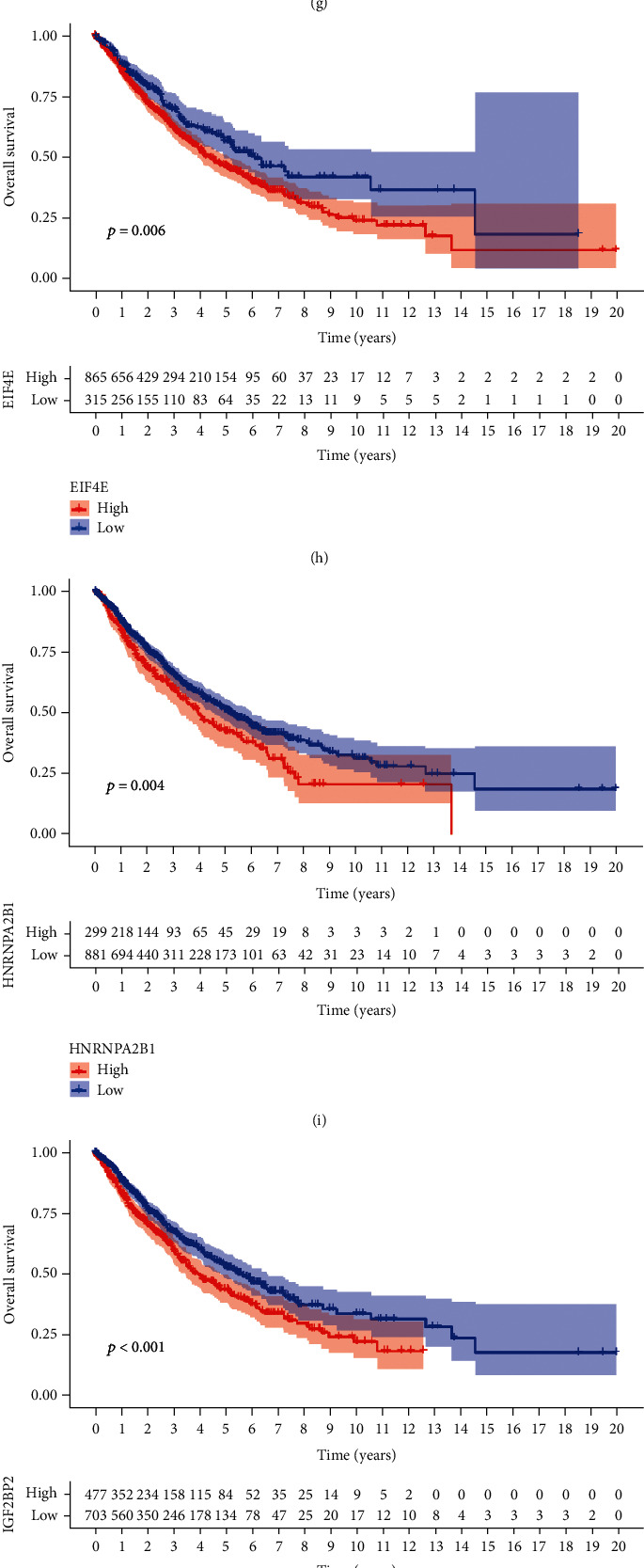
Patterns of m6A modifications mediated by 20 m6A regulators in NSCLC. (a) Interactions of m6A regulators in NSCLC. The size of each circle represents the survival impact of each m6A regulator. The line connecting the m6A regulator and regulator shows the interaction between them. The thickness of the line represents the strength of the correlation between regulators. The *P* value was calculated by log-rank test. (b) Consensus clustering of NSCLC patients for *k* = 2. (c) Consensus clustering CDF for *k* = 2‐9. (d) The CDF curve of consensus clustering. (e) The tracking plot for *k* = 2 to 9. (f) The *P* value of the Kaplan–Meier curve was 0.025, indicating a significant difference in survival between the two m6A modification modes. The survival prognosis of cluster B was better than that of cluster A.

We found that the three categories of m6A regulators, writers, erasers, and readers, not only had highly correlated expression patterns of the same type but also maintained significant correlations with each other. Therefore, we used the m6A regulator network loop diagram to describe the interconnections and actions of m6A regulators and their prognostic impact on NSCLC patients (Figure 3(a)). In addition, we performed survival analysis of the 20 m6A regulators by first selecting the best “cutoff” by KM analysis to classify them into high and low groups. When the *P* value was <0.05, it indicated that the m6A regulators were correlated with prognosis, as shown in the figure. We selected 8 m6A regulators from the 20 m6A regulators, namely, METTL3, RBM15, HNRNPC, IGF2BP2, IGF2BP3, EIF4E, RBM15B, and HNRNPA2B1, of which METTL3 is a low-risk gene, and its survival with high expression is better than that with low expression. The rest were high-risk genes, and the higher the expression was, the higher the risk (Figures 3(g)–3(n)). However, NSCLC patients were classified according to the gene expression level of m6A regulators, which was divided into two different methylation modification modes (Figures 3(b)–3(e) and Figure S1A-C). We refer to these pattern types as m6A cluster A and m6A cluster B. The Kaplan–Meier plot shows significant survival variability between the two m6A modification patterns, with m6A cluster B having a particularly significant survival advantage.

### 3.3. Enrichment Analysis of Different m6A Methylation Modification Patterns and Immune Cell Infiltration

**Figure 4:**
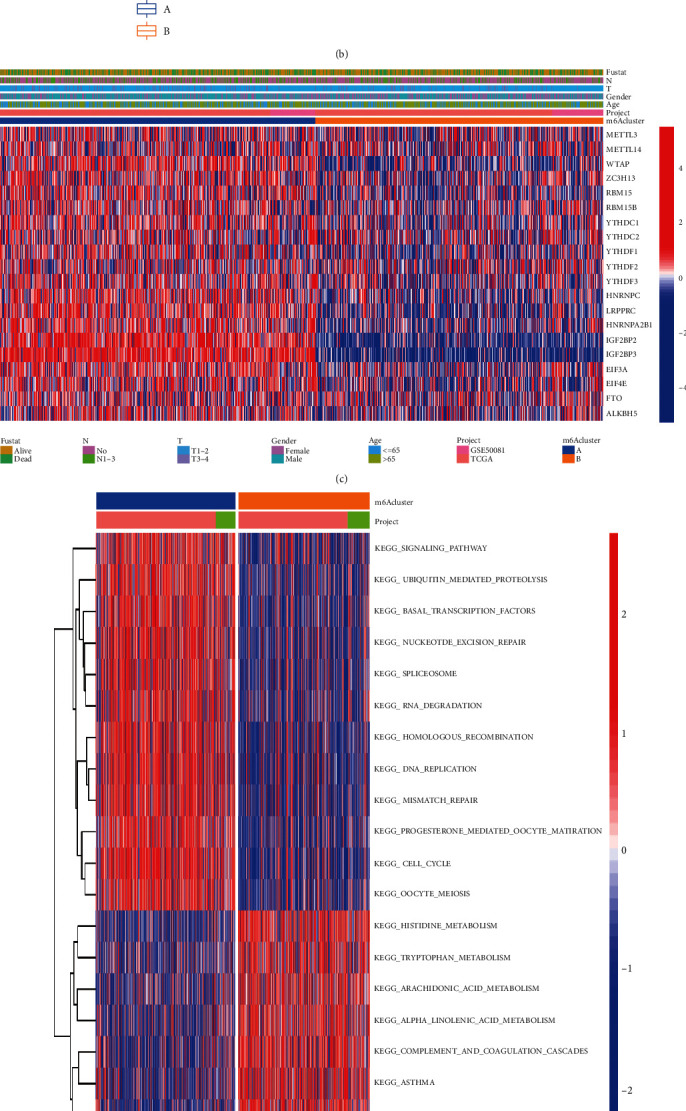
Enrichment analysis of different m6A methylation modification patterns and immune cell infiltration. (a) NSCLC was divided into two different methylation modification patterns using PCA. (b) Infiltration levels of 23 immune cell types in cluster 1/2 in NSCLC (^∗^*P* < 0.05; ^∗∗^*P* < 0.01; ^∗∗∗^*P* < 0.001). (c) Comparison of the relationship between the two clustered clinicopathologies. (d) GSVA enrichment revealed the activation of various pathways in different m6A modification modes. (e) Functional annotation of GO enrichment analysis.

Based on previous studies, we found that the m6A regulator often makes a difference in multiple biological functions, and it plays a large role in cancer, such as proliferation, migration, and invasion [17]. m6A is also involved in cell fate determination, cell cycle regulation, and cell differentiation processes [28]. We used PCA to separate them into two different methylation modification patterns (Figure 4(a)). Then, we used GSVA enrichment. As shown (Figure 4(d) and Table S1), m6A cluster A was significantly enriched in oncogenic pathways and the cell cycle, including the p53 signaling pathway, ubiquitin-mediated proteolytic pathway, nucleotide excision repair, spliceosome, RNA degradation, homologous recombination, DNA replication, mismatch repair, progesterone-mediated oocyte maturation, cell cycle, and oocyte meiosis. m6A cluster B presented enriched pathways significantly associated with nucleotide metabolic pathways, including histidine metabolism, tryptophan metabolism, arachidonic acid metabolism, alpha-linolenic acid metabolism, sulfur metabolism, complement and coagulation systems, and primary biological acid biosynthesis. Further ssGSEA showed that m6A cluster B had a higher level of infiltration in immune cells than m6A cluster A (Figure 4(b)). According to the above analysis, we hypothesized that it has different immune subtypes with different immune mechanisms and utility, confirming the reliability of our study. In addition, we also calculated the DEGs between clusters and annotated them with GO and KEGG functions to further investigate their biological functions (Figure 4(e) and Figure S1D-E), and a significant correlation was found.

### 3.4. m6A Gene Signature Isoforms and m6Ascore Performance Validation

**Figure 5:**
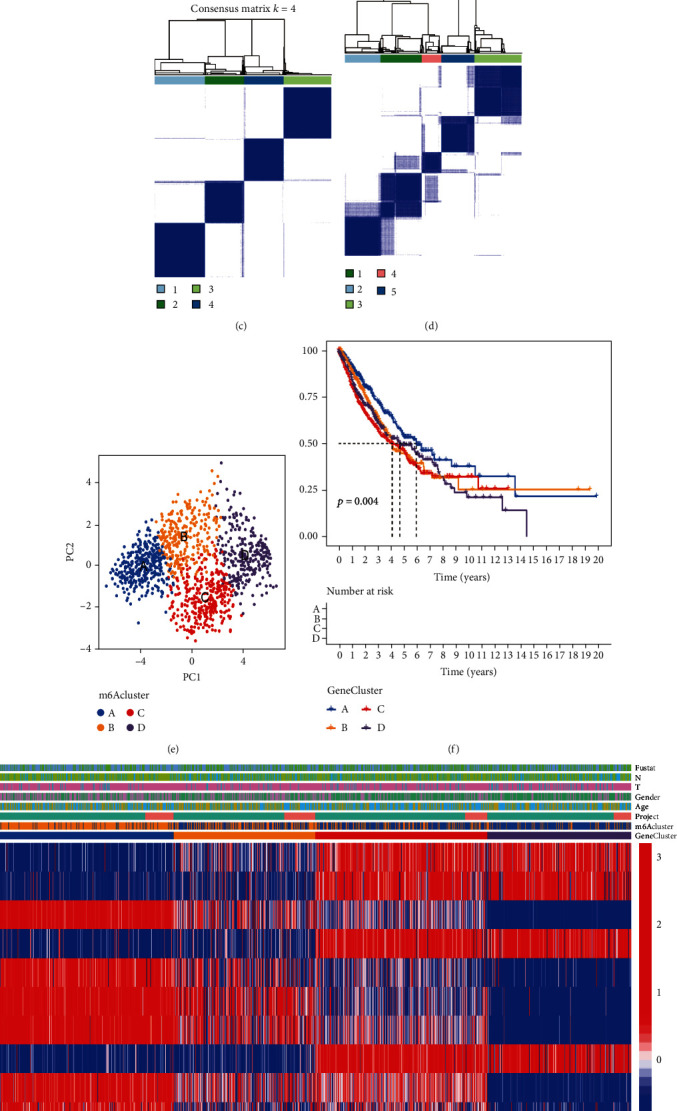
m6A gene signature subtypes and m6Ascore performance validation. (a–d) Consensus clustering of NSCLC patients for *k* = 2‐5. (e) PCA was used to divide the intersecting genes of two different m6A modification patterns into four different molecular subtypes. (f) This Kaplan–Meier curve plot with a *P* value < 0.001 shows that significant gene cluster A had significantly better overall survival than the others. (g) Unsupervised clustering of the intersecting genes of two different m6A methylation modification patterns was used to divide patients into four subtypes, known as m6A gene clusters A-D, where survival, age, sex, and tumor staging were used as reference indicators.

**Figure 6:**
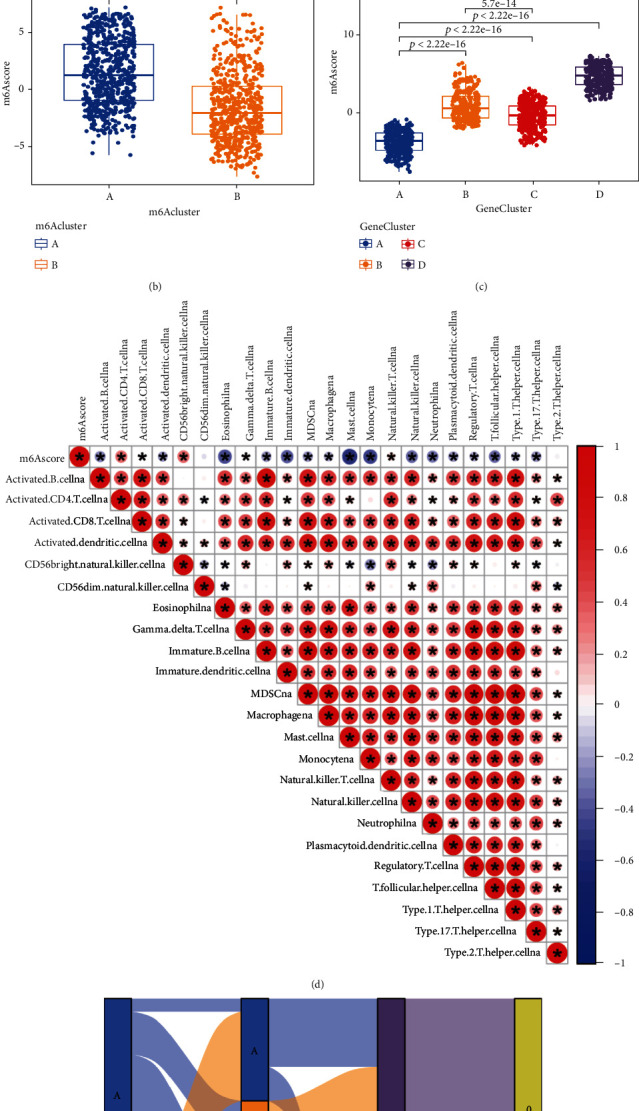
(a) Expression of 20 m6A regulators in the 4 m6A gene clusters. (b) Differences in m6Ascores between the 4 m6A clusters. The Kruskal–Wallis test was used for significant differences. (c) Differences in m6Ascore between the 4 m6A gene clusters (Kruskal–Wallis test, *P* < 0.001). (d) Correlation of m6Ascore with known gene features in the database using Spearman's calculus. Positive correlations are marked in red, and negative correlations are marked in blue. (e) Sankey diagram showing the relationship between m6A cluster, gene cluster, m6Ascore, and survival (0: alive; 1: dead).

Considering the mutations and potential biological functions in NSCLC, we further explored them. Differential analysis using the “Limma” R package identified differentially expressed genes between two different m6A clusters. Then, we selected genes with *P* < 0.001 in univariate Cox regression analysis, leaving 15 genes as DEGs (Figure S2A and Table S2). To further investigate the mechanism of action of these intersecting genes, we performed unsupervised cluster analysis and classified them into four different subtypes (Figures 5(a)–5(d) and Figure S2B-D) and named them m6A gene clusters A-D, where survival, age, sex, and tumor stage were used as reference indicators. The same effect could be obtained for verification using PCA (Figure 5(e)). The results showed that cluster A had a significant survival advantage. The worst results were obtained for cluster C (Figure 5(f)). Unsupervised clustering was performed on the crossover genes of two different m6A methylation modification patterns, and the patients were divided into 4 subtypes. Heatmaps were drawn using survival rate, age, sex, and tumor stage as reference indices, and significant significance was found (Figure 5(g)). There were significant differences in m6A regulators among the four different clusters (Figure 6(a)), and the results once again proved that the m6A methylation pattern is closely related to the occurrence and development of NSCLC.

Subsequently, we built a new evaluation method, which we called m6Ascore. To better characterize the m6A gene clusters, we analyzed the relevance between the m6Ascore and some biological functions (Figure 6(d)). The variation in the properties of individual NSCLC patients can be shown by Sankey diagrams (Figure 6(e)). In addition, the Kruskal–Wallis test proved that the m6Ascore was different in m6A clusters and m6A gene clusters (Figures 6(b) and 6(c)).

**Figure 7:**
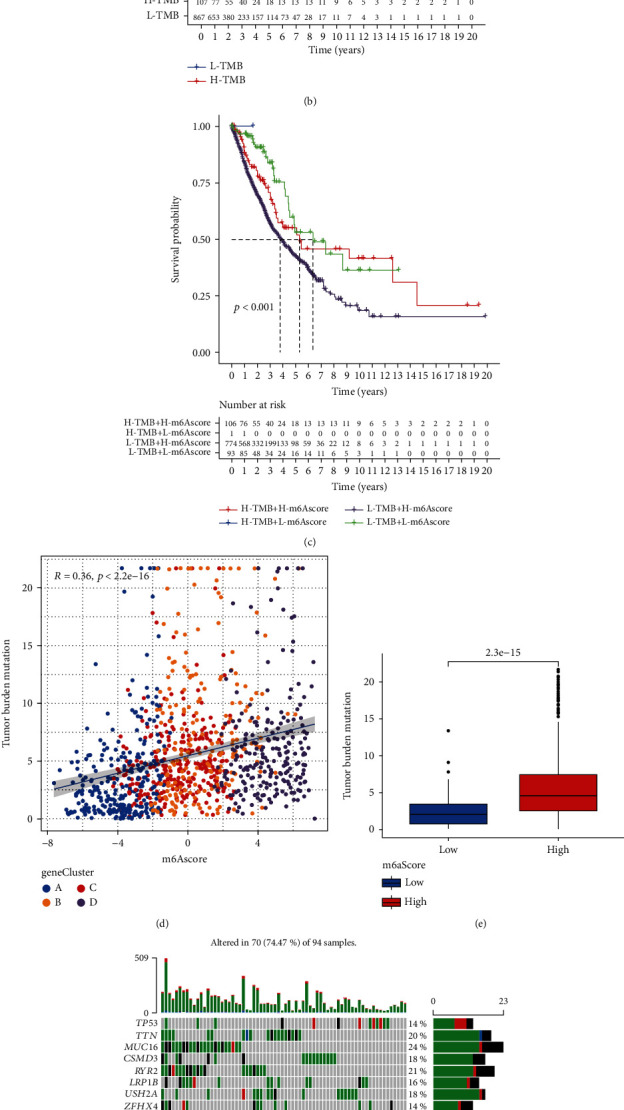
(a) Kaplan–Meier curves depicting survival differences between low and high m6Ascore patient groups. (b) Differential analysis of tumor mutational load between high and low groups in TCGA-NSCLC cohort. (c) Differential analysis of tumor mutational load combined with m6Ascore survival between the high and low groups in TCGA-NSCLC cohort. (d) Correlation analysis of m6Ascore with TBM for different m6A gene clusters. (e) Box plots showing the differential analysis of tumor mutational load (TBM) in the high m6Ascore group or low m6Ascore group. (f, g) Waterfall plots depict differences in the distribution of somatic mutational load in tumors with (f) low m6Ascore and (g) high m6Ascore.

Then, to better explore the prognostic value of NSCLC patients, the “Survminer” R package was used to determine the optimal threshold and divide the total sample amount into high and low groups. Patients with high m6Ascores showed significantly impaired survival (Figure 7(a)). We divided the tumor mutation load in TCGA-NSCLC into H-TBM and L-TBM groups. Differential analyses were performed for the H-TBM and L-TBM groups alone and for the H-TBM and L-TBM groups combined with m6Ascore differential analysis. The picture proved that the survival advantage of H-TBM was greater than that of L-TBM (Figures 7(b) and 7(c)). Correlation analysis of m6Ascore with TBM for different m6A gene clusters showed a significant positive correlation (Figure 7(d)). The box plot shows that the group with a higher m6Ascore had a higher TMB (Figure 7(e)). We used the “Maftools” R package to draw a waterfall diagram and analyze differences between somatic mutations with low and high m6Ascore (Figures 7(f) and 7(g)). The high m6Ascore group had more extensive expression.

### 3.5. Role of m6A Modification Patterns in IPS and Anti-PD-1/L1 Immunotherapy

**Figure 8:**
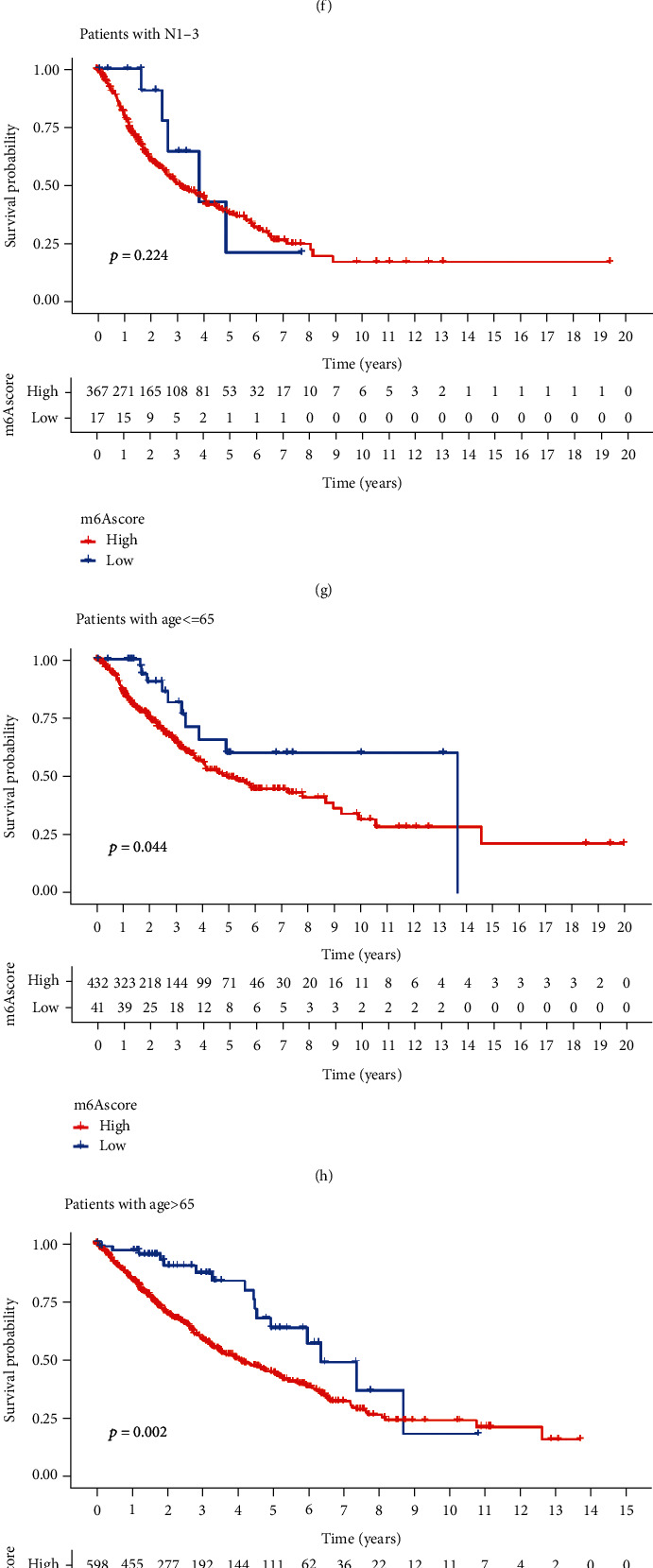
Role of m6A modification patterns in IPS and anti-PD-1/L1 immunotherapy. (a) Wilcoxon test showed differences in PD-L1 expression between the two assessment subgroups. (b, c) Box plots and histograms showing prognostic analysis of survival in the high m6Ascore group or low m6Ascore group (0: alive; 1: dead). (d–i) Kaplan–Meier graphs depict differences in clinical relevance between the two assessment subgroups, including age and T and N stages. (j–m) Immunotherapy scoring for the low m6Ascore group and the high m6Ascore group (po: positive; neg: negative).

Anti-PD-1/PD-L1 therapies have been a popular treatment option in oncology. First, the Wilcoxon test was used to find that PD-L1 expression was different between the low m6Ascore group and the high m6Ascore group, and the high m6Ascore group had higher PD-L1 expression (Figure 8(a)). According to the survival and prognosis analysis of the two groups, m6Ascore was highly expressed in patients with high mortality (Figure 8(b)). The survival rate was higher than the mortality rate in the low m6Ascore group (Figure 8(c)). In the clinical correlation analysis, T and N stages and age were selected as reference indices, and it was found that the low m6Ascore group had a higher overall survival rate (Figures 8(d)–8(i)). In the immunotherapy scoring, we found significant differences between the high m6Ascore group and the low m6Ascore group in all four immunotherapy regimens, and it is worth mentioning that anti-PD-1(+) is more effective in the low m6Ascore group in IPS. A low m6Ascore score indicates a better therapeutic effect of anti-PD-1/PD-L1 therapies (Figures 8(j)–8(m)). In summary, m6A methylation modification patterns are associated with anti-PD-1/L1 immunotherapy and will help to predict the response to anti-PD-1/L1 immunotherapy.

## 4. Discussion

Lung cancer is currently one of the most common malignant tumors around the world with a high mortality rate [29], and NSCLC is a fatal malignant tumor that has poor prognosis, accounting for 85-90% of the total incidence of lung cancer [30–32]. With the continuous progress of medical technology, great progress has been made in the diagnosis and treatment of NSCLC. However, due to the imperfection of screening tests and the late emergence of clinical symptoms, effective diagnosis and prognosis treatment for NSCLC patients to greatly improve the survival rate is still a huge challenge [33]. Therefore, we need to further improve the level of diagnosis and treatment of NSCLC.

Previous evidence suggests that m6A modification patterns play an indispensable role in the tumor immune microenvironment and antitumor therapy through interactions with various m6A regulators [34, 35]. The m6A regulators selected in this paper are shown in Table 1. Previous studies have shown that m6A RNA methylation has a significant impact on the development and progression of NSCLC and the tumor microenvironment [36]. For example, YTHDF1 enhances the antitumor response of tumor-infiltrating CD8+ T cells, thereby promoting tumorigenesis and progression and leading to poor prognosis [11, 20]. FTO activates cell migration through m6A demethylation, thereby promoting lung cancer cell progression [37]. METTL3 enhances mRNA translation through the interaction of translation initiation machinery, thereby promoting human lung cancer cell growth, survival, and invasion [38, 39]. Meanwhile, specific depletion of METTL3 or METTL14 enhanced the efficacy of anti-PD-L1 therapy [40]. IGF2BP3 regulates the interaction with miRNAs through a variety of mechanisms, thereby affecting the expression of malignancy-associated RNAs [41]. ALKBH5 is also an independent prognostic indicator for multiple cancers, is significantly upregulated in NSCLC tissues and cells, and contributes to the malignant features of NSCLC cells by suppressing TIMP3 mRNA stability dependent on m6A demethylation modifications [42, 43]. Because most studies have been limited to the m6A regulators themselves, we explored the biological functions of the modification patterns of m6A regulators in NSCLC in a holistic manner.

In this text, we used consensus classification to divide patients into two different m6A clusters based on the mRNA expression levels of 20 m6A regulators. These two different clusters have different clinical features, prognostic value, and immune cell infiltration. Patients in the m6A cluster B group had a better prognosis than those in the m6A cluster A group. Considering that the high probability of prognosis is related to the tumor immune microenvironment, we continued to analyze the difference in immune infiltration between m6A cluster A and m6A cluster B and found higher levels in m6A cluster B. The m6A cluster classification based on immunophenotype is reliable. To further explore the potential biological functions between the two clusters, we selected fifteen DEGs and performed GO and KEGG enrichment analysis to analyze their biological functions; notably, the immune-related biological processes were significantly associated with them. Meanwhile, we divided four m6A gene clusters based on DEGs. The prognostic value and clinical features showed that the m6A gene cluster was closely related to the occurrence and development of NSCLC. Considering the heterogeneity of m6A modification, it is necessary to quantify the m6A modification pattern of individual tumors. Finally, we built a method to evaluate this pattern, which we call the m6Ascore. The survival advantage of the low m6Ascore group was significantly greater than that of the high m6Ascore group. Meanwhile, the expression level of TBM in the high m6Ascore group was higher than that in the low m6Ascore group. There was a significant positive correlation between m6Ascore and TBM in different m6A gene clusters. m6Ascore is closely associated with immune cell infiltration and tumor mutational load and can be used as a prognostic marker in NSCLC. To date, some studies have confirmed that the m6Ascore is closely related to tumor progression, the tumor microenvironment, and immune cell infiltration. For example [27, 44], based on m6A regulators expressed at the mRNA level in hepatocellular carcinoma and gastric cancer, they were classified into three different isoforms, followed by assessment of clinical features, immune cell infiltration, functional annotation, and prognosis. The success of these studies can serve as a reference. We continued to divide the 15 DEGs into four subtypes to construct the m6Ascore and further analyzed its clinical characteristics, prognosis, immunophenotype, and immunotherapy response.

The discovery of anti-PD-1/L1 therapy has led to improved outcomes in a variety of advanced cancers, including NSCLC [45]. Notably, although it has many advantages in clinical treatment, the outcome of immunotherapy shows strong individual variability [46], and checkpoint inhibition immune checkpoint therapy is the most clinically studied and widely used tumor immunotherapy. It is a therapeutic approach that inhibits tumor cell progression by synergistically activating or inhibiting T cell activity. We detected the expression levels of PD-1/L1 and CTLA-4 in NSCLC. The expression level of PD-L1 was higher in the high m6Ascore group, and the difference in IPS among the three groups was statistically significant. The results indicated a significant correlation between m6Ascore and the efficacy of immunotherapy. A low m6Ascore indicates a better therapeutic effect of anti-PD-1/PD-L1 therapies. The m6Ascore scoring mechanism has good predictive value in anti-PD-L1 immunotherapy.

Overall, we classified m6A regulators into two distinct subtypes based on their mRNA expression in NSCLC, followed by a comprehensive assessment of clinical features, immune cell infiltration, and prognostic value. The screened differential genes were further classified into four subtypes, and we further analyzed clinical features, immunophenotyping, and immunotherapy by constructing a m6Ascore. m6A modification was related to the tumor microenvironment and immune cell infiltration. Meanwhile, checkpoint inhibition immune checkpoint therapy has some efficacy in NSCLC. However, this paper has some limitations that we need to consider. Since our study was limited to public databases, we need further experiments to confirm our results. In addition, more studies should be applied to confirm the exact mechanism of m6A regulators.

## 5. Conclusion

Studies have shown that m6A RNA methylation patterns make a large difference in the tumor immune microenvironment of NSCLC. Copy number variation, mRNA expression levels, tumor microenvironment, immune cell infiltration, potential biological functions, clinical prognostic value, and immunotherapeutic response were comprehensively evaluated. m6A modification was associated with the tumor microenvironment, as well as immune cell infiltration. The m6Ascore can predict the efficacy of anti-PD-L1 immunotherapy. A low m6Ascore indicates a better therapeutic effect of anti-PD-1/PD-L1 therapies. Our findings provide new strategies to promote individualized tumor immunotherapy and potential therapeutic targets for NSCLC.

## Figures and Tables

**Table 1:** Twenty m6A RNA methylation regulators were selected in this study.

Regulators	Full name	Type
METTL3	Methyltransferase like 3	Writers
METTL14	Methyltransferase like 14	Writers
WTAP	WT1-associated protein	Writers
ZC3H13	Zinc finger CCCH-type containing 13	Writers
RBM15	RNA-binding motif protein 15	Writers
RBM15B	RNA-binding motif protein 15B	Writers
YTHDC1	YTH domain containing 1	Readers
YTHDC2	YTH domain containing 2	Readers
YTHDF1	YTH N6-methyladenosine RNA-binding protein 1	Readers
YTHDF2	YTH N6-methyladenosine RNA-binding protein 2	Readers
YTHDF3	YTH N6-methyladenosine RNA-binding protein 3	Readers
HNRNPC	Heterogeneous nuclear ribonucleoprotein C	Readers
LRPPRC	Leucine-rich pentatricopeptide repeat containing	Readers
HNRNPA2B1	Heterogeneous nuclear ribonucleoprotein A2B1	Readers
IGF2BP2	IGF2 mRNA-binding protein 2	Readers
IGF2BP3	IGF2 mRNA-binding protein 3	Readers
EIF3A	Eukaryotic translation initiation factor 3 subunit A	Readers
EIF4E	Eukaryotic translation initiation factor 4 subunit E	Readers
FTO	Fat mass and obesity-associated protein	Erasers
ALKBH5	*α*-Ketoglutarate-dependent dioxygenase AlkB homolog 5	Erasers

## Data Availability

The datasets downloaded for supporting the results of this article are publicly available at TCGA (https://portal.gdc.cancer.gov/) and GEO (https://www.ncbi.nlm.nih.gov/geo/).

## Abbreviations

NSCLC: Non-small-cell lung cancer
TME: Tumor microenvironment
CNV: Copy number variation
GSVA: Gene set variation analysis
PCA: Principal component analysis
ssGSEA: Single-sample gene-set enrichment analysis
TCGA: The Cancer Genome Atlas
GEO: Gene Expression Omnibus
GO: Gene ontology
DEGs: Differentially expressed genes
HR: Hazard ratio.

## Supplementary Materials

Supplementary Materials Figure S1: (A–C) consensus clustering of NSCLC patients for *k* = 3‐5. (D, E) Functional annotation of GO and KEGG enrichment analysis. Figure S2: (A) univariate Cox regression analysis of fifteen DEGs. (B) Consensus clustering CDF for *k* = 2‐9. (C) The CDF curve of consensus clustering. (D) The tracking plot for *k* = 2 to 9. Table S1: activation states of biological pathways in distinct m6A modification patterns by GSVA enrichment. Table S2: univariate Cox regression analysis of 15 m6A-related genes.
