# Supplementary material for: Integrative Analysis of m6A RNA Methylation Regulators and the Tumor Immune Microenvironment in Non-Small-Cell Lung Cancer
Source: Dis Markers. 2022 Feb 9;2022:2989200. doi: 10.1155/2022/2989200 (PMC8849944; doi:10.1155/2022/2989200)
Supplement: Supplementary Materials — Figure S1: (A–C) consensus clustering of NSCLC patients for k = 3‐5. (D, E) Functional annotation of GO and KEGG enrichment analysis. Figure S2: (A) univariate Cox regression analysis of fifteen DEGs. (B) Consensus clustering CDF for k = 2‐9. (C) The CDF curve of consensus clustering. (D) The tracking plot for k = 2 to 9. Table S1: activation states of biological pathways in distinct m6A modification patterns by GSVA enrichment. Table S2: univariate Cox regression analysis of 15 m6A-related genes. [file 2989200.f1.zip › 2989200.f1/Revision TableS1.pdf]

TableS1:Activation states of biological pathways in distinct m6A modification patterns by GSVA enrichment analysis

PATHWAY	logFC	AveExpr	t	P.Value	adj.P.Val	B
KEGG CELL CYCLE	-0.394756	-0.015491845	-26.26660038	1.57E-120	2.89E-118	264.4436118
KEGG_PROGESTERONE_MEDIATED_OOCYTE_MATURATION	-0.211463	-0.012306936	-23.76362063	1.25E-102	1.15E-100	223.3703551
KEGG PRIMARY BILE ACID BIOSYNTHESIS	0.3533823	0.006920826	23.16647555	1.89E-98	1.16E-96	213.7790281
KEGG_HOMOLOGOUS_RECOMBINATION	-0.414562	-0.024528619	-22.5049765	7.22E-94	3.32E-92	203.2637608
KEGG_OOCYTE_MEIOSIS	-0.217965	-0.010952174	-22.20862777	7.84E-92	2.89E-90	198.5927961
KEGG BASAL TRANSCRIPTION FACTORS	-0.32523	-0.018404203	-22.05667634	8.59E-91	2.63E-89	196.2077636
KEGG_HISTIDINE_METABOLISM	0.2585656	-0.004401534	21.8918168	1.14E-89	3.01E-88	193.627962
KEGG DNA REPLICATION	-0.467374	-0.015597659	-21.73082479	1.42E-88	3.27E-87	191.1166966
KEGG_MISMATCH_REPAIR	-0.410921	-0.023684299	-21.66518064	3.97E-88	8.11E-87	190.0950404
KEGG UBIQUITIN MEDIATED PROTEOLYSIS	-0.237306	-0.019201163	-21.56103678	2.01E-87	3.71E-86	188.4769678
KEGG_NUCLEOTIDE_EXCISION_REPAIR	-0.320242	-0.02490063	-20.24105378	1.29E-78	2.15E-77	168.2802448
KEGG_COMPLEMENT_AND_COAGULATION_CASCADES	0.3275879	0.024437892	19.97218144	7.41E-77	1.14E-75	164.2415088
KEGG SPLICEOSOME	-0.33134	-0.02184253	-19.78241615	1.28E-75	1.81E-74	161.4073175
KEGG_RNA_DEGRADATION	-0.281355	-0.021041154	-18.421367	6.14E-67	8.07E-66	141.4988142
KEGG ARACHIDONIC ACID METABOLISM	0.2208005	0.016556856	18.14516897	3.23E-65	3.96E-64	137.5538998
KEGG_SULFUR_METABOLISM	0.2925646	-0.002921678	17.70892601	1.57E-62	1.81E-61	131.3926883
KEGG ALPHA LINOLENIC ACID METABOLISM	0.2512011	0.018480724	17.34499555	2.57E-60	2.78E-59	126.3200905
KEGG_ASTHMA	0.3646641	0.017410421	16.86887872	1.84E-57	1.88E-56	119.7797011
KEGG_P53_SIGNALING_PATHWAY	-0.179717	-0.019119824	-16.29092732	4.60E-54	4.45E-53	111.9923787
KEGG_TRYPTOPHAN_METABOLISM	0.1797881	0.004467056	15.89530272	8.83E-52	8.12E-51	106.761646
KEGG_RENAL_CELL_CARCINOMA	-0.174257	-0.008010388	-15.32856109	1.42E-48	1.25E-47	99.41580103
KEGG BASE EXCISION REPAIR	-0.257284	-0.022158174	-15.00303258	9.13E-47	7.63E-46	95.27750185
KEGG_SMALL_CELL_LUNG_CANCER	-0.156906	-0.009583427	-14.76626282	1.81E-45	1.45E-44	92.30575479
KEGG PPAR SIGNALING PATHWAY	0.1416045	0.010218015	14.45264718	9.03E-44	6.92E-43	88.42008592
KEGG_NICOTINATE_AND_NICOTINAMIDE_METABOLISM	0.175381	-0.004382331	14.35900635	2.87E-43	2.11E-42	87.27123341
KEGG PANCREATIC CANCER	-0.146587	-0.013603581	-14.15744837	3.39E-42	2.40E-41	84.81633667
KEGG_CHRONIC_MYELOID_LEUKEMIA	-0.148268	-0.01136892	-14.00228719	2.23E-41	1.52E-40	82.94341673
KEGG_AUTOIMMUNE_THYROID_DISEASE	0.2910186	0.03065409	13.95193915	4.10E-41	2.70E-40	82.33886106
KEGG INTESTINAL IMMUNE NETWORK FOR IGA PRODUCTION	0.2934344	0.008883748	13.74722511	4.79E-40	3.04E-39	79.89697602
KEGG_HEMATOPOIETIC_CELL_LINEAGE	0.2423778	0.016090018	13.35292229	5.06E-38	3.10E-37	75.26809358
KEGG LINOLEIC ACID METABOLISM	0.199253	0.020238375	13.28734216	1.09E-37	6.45E-37	74.50787049
KEGG_PROSTATE_CANCER	-0.123319	-0.008292411	-13.24916991	1.69E-37	9.74E-37	74.06664813
KEGG NITROGEN METABOLISM	0.1650922	0.003771965	13.24424386	1.79E-37	1.00E-36	74.00977818
KEGG_ALLOGRAFT_REJECTION	0.3310346	0.008815083	13.23094914	2.09E-37	1.13E-36	73.85637248
KEGG_PROTEASOME	-0.279016	-0.020225041	-13.14435082	5.71E-37	3.00E-36	72.85994275
KEGG GLIOMA	-0.130468	-0.007112167	-12.95044655	5.30E-36	2.71E-35	70.64661854
KEGG_LYSINE_DEGRADATION	-0.155138	-0.028282711	-12.90340715	9.08E-36	4.51E-35	70.11342231
KEGG GLYCOSPHINGOLIPID BIOSYNTHESIS GANGLIO SERIES	0.2012679	0.010327153	12.8167798	2.43E-35	1.18E-34	69.13533585
KEGG_SYSTEMIC_LUPUS_ERYTHEMATOSUS	0.2404553	0.019669312	12.58910758	3.16E-34	1.49E-33	66.58867847
KEGG TYPE I DIABETES MELLITUS	0.2718838	0.011019579	12.52968065	6.14E-34	2.82E-33	65.9297005
KEGG_RNA_POLYMERASE	-0.211552	-0.018582189	-12.14662537	4.18E-32	1.88E-31	61.73995054
KEGG_ONE_CARBON_POOL_BY_FOLATE	-0.194078	-0.023243974	-12.05436805	1.14E-31	4.99E-31	60.74600808
KEGG_FATTY_ACID_METABOLISM	0.1706645	-0.01681585	11.94074107	3.88E-31	1.66E-30	59.5300103
KEGG_CELL_ADHESION_MOLECULES_CAMS	0.1886519	0.016509753	11.84329914	1.10E-30	4.61E-30	58.49444709
KEGG CYTOKINE CYTOKINE RECEPTOR INTERACTION	0.1780732	0.01512579	11.77564218	2.27E-30	9.27E-30	57.77936988
KEGG_ALDOSTERONE_REGULATED_SODIUM_REABSORPTION	0.1359163	0.011963335	11.53965744	2.73E-29	1.09E-28	55.31072191
KEGG PYRIMIDINE METABOLISM	-0.145966	-0.020369561	-11.51481571	3.54E-29	1.39E-28	55.05317277
KEGG_RENIN_ANGIOTENSIN_SYSTEM	0.1843963	0.006452853	11.48974025	4.60E-29	1.76E-28	54.79365121
KEGG_GLYCOSAMINOGLYCAN_DEGRADATION	0.1804383	0.00698433	11.48302797	4.94E-29	1.85E-28	54.72425862
KEGG_TYROSINE_METABOLISM	0.1340913	0.00135805	11.2346142	6.43E-28	2.37E-27	52.17908947
KEGG_ERBB_SIGNALING_PATHWAY	-0.105142	-0.004880104	-11.13713914	1.74E-27	6.28E-27	51.19269152
KEGG MATURITY ONSET DIABETES OF THE YOUNG	0.166214	0.036356753	11.12929913	1.88E-27	6.66E-27	51.11365782
KEGG_NEUROACTIVE_LIGAND_RECEPTOR_INTERACTION	0.122606	0.032851372	11.12755142	1.92E-27	6.66E-27	51.09604557
KEGG AMINOACYL TRNA BIOSYNTHESIS	-0.204566	-0.02524713	-11.10075628	2.52E-27	8.58E-27	50.82630476
KEGG_GRAFT_VERSUS_HOST_DISEASE	0.2744241	0.009653875	11.0384625	4.73E-27	1.58E-26	50.20125341
KEGG TAURINE AND HYPOTAURINE METABOLISM	0.1667928	0.000227865	11.01376839	6.07E-27	2.00E-26	49.95426825
KEGG_PROXIMAL_TUBULE_BICARBONATE_RECLAMATION	0.1403912	0.007346509	10.97925721	8.60E-27	2.77E-26	49.60985161
KEGG_COLORECTAL_CANCER	-0.113166	-0.011198008	-10.80470091	4.92E-26	1.56E-25	47.88137599
KEGG BLADDER CANCER	-0.114181	-0.01758897	-10.68929286	1.54E-25	4.65E-25	46.75110949
KEGG_ARGININE_AND_PROLINE_METABOLISM	0.1205869	-0.008781959	10.66798165	1.90E-25	5.64E-25	46.54349068
KEGG VIRAL MYOCARDITIS	0.1678468	0.011402456	10.41282477	2.28E-24	6.65E-24	44.08440301
KEGG_PEROXISOME	0.1222565	-0.017130129	10.32804	5.14E-24	1.48E-23	43.27826679
KEGG O GLYCAN BIOSYNTHESIS	0.1456775	0.008485216	10.32112422	5.49E-24	1.55E-23	43.21275438
KEGG_ANTIGEN_PROCESSING_AND_PRESENTATION	0.1805917	0.013206701	10.27337193	8.66E-24	2.41E-23	42.76140526
KEGG_NON_SMALL_CELL_LUNG_CANCER	-0.100353	-0.010321876	-10.18462481	2.01E-23	5.45E-23	41.92724256
KEGG LYSOSOME	0.1472957	-0.005429007	10.04651163	7.38E-23	1.97E-22	40.64119362
KEGG_ADHERENS_JUNCTION	-0.117333	-0.005945235	-9.851288543	4.52E-22	1.19E-21	38.84871278
KEGG BETA ALANINE METABOLISM	0.1168628	-0.004807266	9.825887681	5.71E-22	1.48E-21	38.61768544
KEGG_OTHER_GLYCAN_DEGRADATION	0.1711856	0.002771941	9.779520117	8.74E-22	2.20E-21	38.19727013
KEGG THYROID CANCER	-0.11138	-0.016875749	-9.712384734	1.61E-21	4.01E-21	37.59155816
KEGG_STEROID_HORMONE_BIOSYNTHESIS	0.1229749	0.021055186	9.658609381	2.63E-21	6.36E-21	37.10895494
KEGG_LEUKOCYTE_TRANSENDOTHELIAL_MIGRATION	0.1228998	0.012102886	9.30884886	5.94E-20	1.40E-19	34.02628376
KEGG_TASTE_TRANSDUCTION	0.1066181	0.037442489	9.298184334	6.53E-20	1.52E-19	33.93383146
KEGG_VASCULAR_SMOOTH_MUSCLE_CONTRACTION	0.1013222	0.008528154	9.283221965	7.44E-20	1.71E-19	33.80427519
KEGG NON HOMOLOGOUS END JOINING	-0.157179	-0.019843491	-9.22686159	1.22E-19	2.77E-19	33.3178847
KEGG_ENDOMETRIAL_CANCER	-0.101184	-0.005712446	-8.967238997	1.14E-18	2.56E-18	31.11063066
KEGG JAK STAT SIGNALING PATHWAY	0.1032909	0.016249066	8.698895441	1.08E-17	2.40E-17	28.88722663
KEGG_CITRATE_CYCLE_TCA_CYCLE	-0.157641	-0.016101136	-8.674158448	1.33E-17	2.92E-17	28.68525592
KEGG DRUG METABOLISM CYTOCHROME P450	0.1332316	0.01217937	8.399919513	1.25E-16	2.67E-16	26.48027865
KEGG_PENTOSE_PHOSPHATE_PATHWAY	-0.128712	-0.003803036	-8.285244674	3.12E-16	6.59E-16	25.57691304
KEGG_ASCORBATE_AND_ALDARATE_METABOLISM	0.1388084	0.012844239	8.220674293	5.20E-16	1.09E-15	25.07312461
KEGG HYPERTROPHIC CARDIOMYOPATHY HCM	0.1000515	0.015020314	8.127553828	1.08E-15	2.23E-15	24.35278919
KEGG_RIBOFLAVIN_METABOLISM	0.111969	-0.009065775	7.791327721	1.43E-14	2.89E-14	21.81331093
KEGG LEISHMANIA INFECTION	0.1368843	0.008330565	7.710557777	2.62E-14	5.23E-14	21.21768749
KEGG_NATURAL_KILLER_CELL_MEDIATED_CYTOTOXICITY	0.1003419	0.0091308	6.970191082	5.21E-12	9.78E-12	16.02214628
KEGG GLYCOSYLPHOSPHATIDYLINOSITOL GPI ANCHOR BIOSYNTHESIS	-0.111019	-0.024976589	-6.839005911	1.27E-11	2.35E-11	15.15180545
KEGG_GLYOXYLATE_AND_DICARBOXYLATE_METABOLISM	-0.109329	-0.019907106	-6.550457065	8.49E-11	1.52E-10	13.29139593
KEGG_PHENYLALANINE_METABOLISM	0.100475	-0.009202284	6.5359796	9.32E-11	1.65E-10	13.20001665
KEGG PRIMARY IMMUNODEFICIENCY	0.1366597	0.000675495	6.13021657	1.19E-09	1.95E-09	10.71582025
