# Supplementary material for: Integrative Analysis of m6A RNA Methylation Regulators and the Tumor Immune Microenvironment in Non-Small-Cell Lung Cancer
Source: Dis Markers. 2022 Feb 9;2022:2989200. doi: 10.1155/2022/2989200 (PMC8849944; doi:10.1155/2022/2989200)
Supplement: Supplementary Materials — Figure S1: (A–C) consensus clustering of NSCLC patients for k = 3‐5. (D, E) Functional annotation of GO and KEGG enrichment analysis. Figure S2: (A) univariate Cox regression analysis of fifteen DEGs. (B) Consensus clustering CDF for k = 2‐9. (C) The CDF curve of consensus clustering. (D) The tracking plot for k = 2 to 9. Table S1: activation states of biological pathways in distinct m6A modification patterns by GSVA enrichment. Table S2: univariate Cox regression analysis of 15 m6A-related genes. [file 2989200.f1.zip › 2989200.f1/Revision TableS2.pdf]

TableS2: Univariate Cox regression analysis of 15 m6A related genes

Gene	HR	HR.95L	HR.95H	pvalue
SLC2A1	1.077045864	1.028251614	1.128155577	0.001702528
FAM83B	1.046886906	1.000117503	1.095843428	0.04941392
C16orf89	0.956594142	0.927371696	0.986737417	0.005056336
SERPINB5	1.03510487	1.003866389	1.067315436	0.027329463
CYP2B7P	0.946493966	0.911870597	0.982431971	0.003826336
SFTA3	0.964140356	0.932916709	0.996409022	0.02969468
NAPSA	0.971556865	0.945873317	0.997937805	0.034773688
KRT6A	1.020630996	1.001320453	1.040313944	0.036138681
SFTPB	0.97304383	0.949141389	0.997548211	0.031286299
CYP4B1	0.942660018	0.907580369	0.97909556	0.002274746
PIGR	0.955855803	0.925703914	0.986989794	0.005767119
FGFBP1	1.034153126	1.000062148	1.069406226	0.049576767
PGC	0.967405853	0.941078863	0.994469348	0.01857614
SCGB3A1	0.952779029	0.925630039	0.980724307	0.001039545
SCGB3A2	0.96552352	0.939053534	0.99273964	0.013370669
