# Supplementary figures and images for: Integrative Analysis of m6A RNA Methylation Regulators and the Tumor Immune Microenvironment in Non-Small-Cell Lung Cancer
Source: Dis Markers. 2022 Feb 9;2022:2989200. doi: 10.1155/2022/2989200 (PMC8849944; doi:10.1155/2022/2989200)

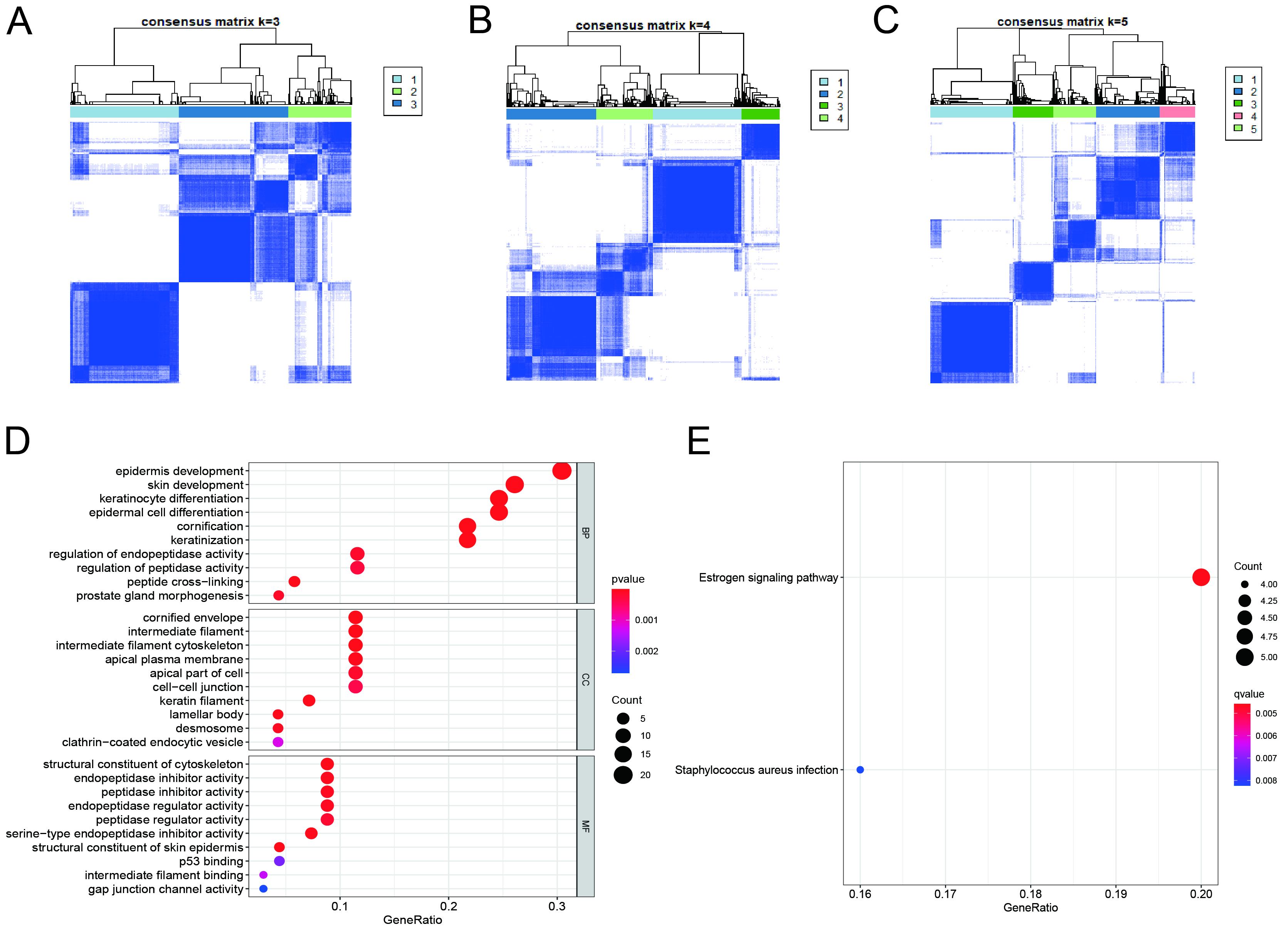

Supplement: Supplementary Materials — Figure S1: (A–C) consensus clustering of NSCLC patients for k = 3‐5. (D, E) Functional annotation of GO and KEGG enrichment analysis. Figure S2: (A) univariate Cox regression analysis of fifteen DEGs. (B) Consensus clustering CDF for k = 2‐9. (C) The CDF curve of consensus clustering. (D) The tracking plot for k = 2 to 9. Table S1: activation states of biological pathways in distinct m6A modification patterns by GSVA enrichment. Table S2: univariate Cox regression analysis of 15 m6A-related genes. [file 2989200.f1.zip › 2989200.f1/FigureS1.jpg]

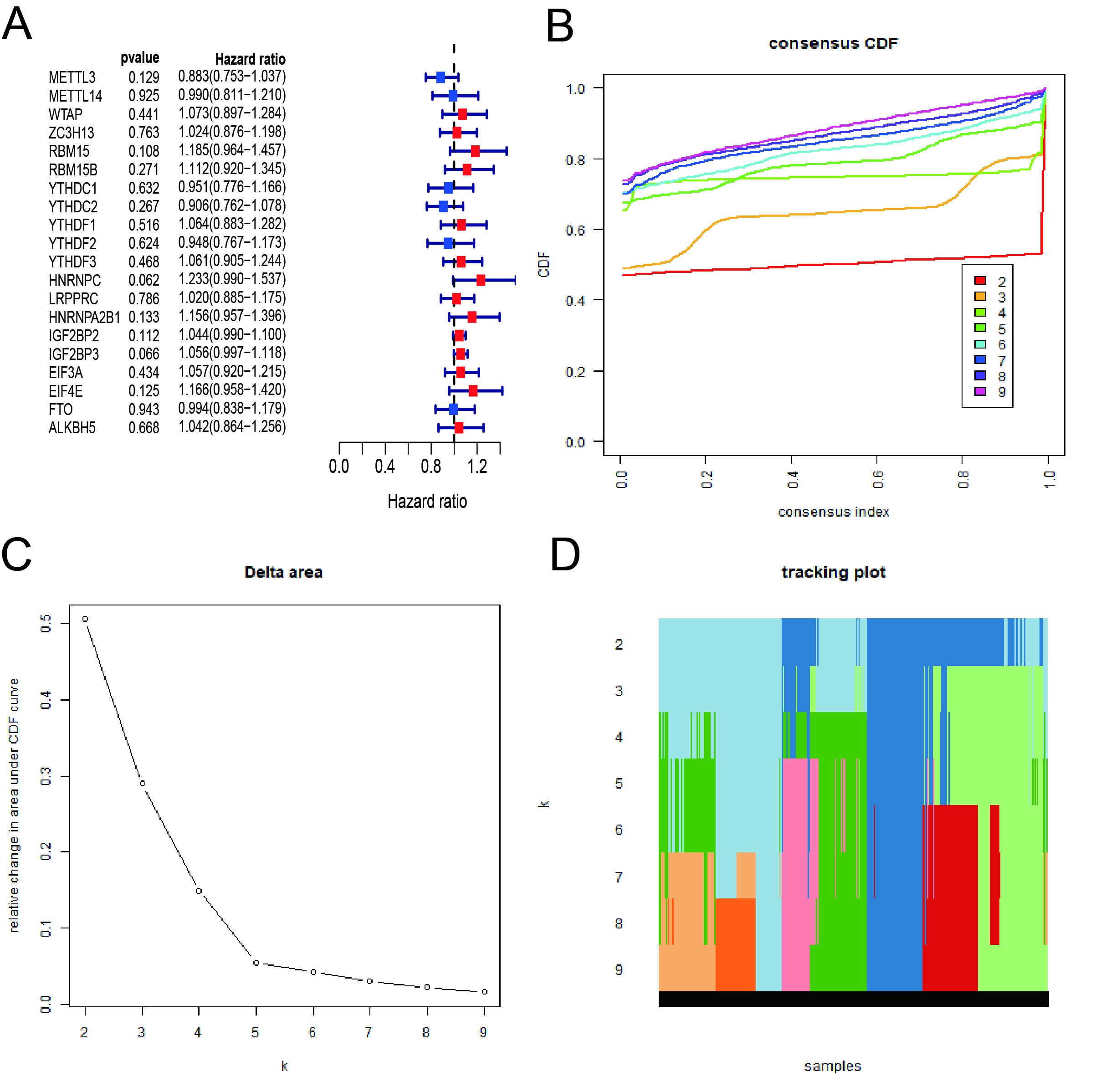

Supplement: Supplementary Materials — Figure S1: (A–C) consensus clustering of NSCLC patients for k = 3‐5. (D, E) Functional annotation of GO and KEGG enrichment analysis. Figure S2: (A) univariate Cox regression analysis of fifteen DEGs. (B) Consensus clustering CDF for k = 2‐9. (C) The CDF curve of consensus clustering. (D) The tracking plot for k = 2 to 9. Table S1: activation states of biological pathways in distinct m6A modification patterns by GSVA enrichment. Table S2: univariate Cox regression analysis of 15 m6A-related genes. [file 2989200.f1.zip › 2989200.f1/Revision FigureS2.jpg]
